# Classification of Thyroid Peroxidase (TPO) Inhibitors Using Transfer Learning with SMILES Embeddings

**DOI:** 10.1021/acs.chemrestox.6c00058

**Published:** 2026-06-02

**Authors:** Geven Piir, Sulev Sild, Eliana Spilioti, Dimitra Nikolopoulou, Effrosyni Katsanou, Ingrid Langezaal, Uko Maran

**Affiliations:** † Institute of Chemistry, 37546 University of Tartu, Ravila 14a, Tartu 50411, Estonia; ‡ Laboratory of Toxicological Control of Pesticides, Scientific Directorate of Pesticides’ Control and Phytopharmacy, 69064 Benaki Phytopathological Institute (BPI), 8 Stefanou Delta street, Kifissia, Attica GR-14561, Greece; § 99013 European Commission, Joint Research Centre (JRC), via E. Fermi, 2749, TP126, Ispra, Varese I-21027, Italy

## Abstract

Thyroid hormones (THs) regulate many processes in mammals and, therefore, affect every organ in the body. Thyroid peroxidase (TPO) is an essential enzyme for the successful biosynthesis of THs. Although TPO inhibition is a well-documented molecular initiating event (MIE) in thyroid hormone system disruption adverse outcome pathways (AOPs), experimental methods and computational models to assess TPO activity are lacking. Efficient computational new approach methodologies (NAMs) are a viable solution for identifying TPO inhibitors from a large pool of agrochemicals. The aim of this study was to investigate the suitability of SMILES embeddings generated using a specialized language model (SLM) based on a pretrained deep neural network (DNN) for applying a transfer learning approach in the development of quantitative structure–activity relationships for classifying TPO inhibitors. Traditional theoretical molecular descriptors were used for comparison. Two different molecular descriptor sets resulted in Random Forest (RF) models that performed similarly on the training and test sets, while the sensitivity for the external validation set was substantially different between the two models (0.788 vs 0.490). Comparison of the predictions with the TPO inhibition data of the chemicals assessed by EFSA and EU-NETVAL laboratories showed good agreement. At the same time, analysis of experimental data from other sources showed some conflicting estimates. This suggests that further and more precise studies are needed for some compounds. This study advances in silico methodologies by implementing transfer learning for QSAR modeling from text representations (e.g., SMILES) using the pretrained Bidirectional Encoder Representations from Transformers (BERT) architecture. While traditional QSAR approach relies on molecular descriptors, this evaluation shows that model-generated SMILES embeddings can expand the applicability domain, indicating a more robust representation of structural information compared to traditional molecular descriptors.

## Graphical abstract

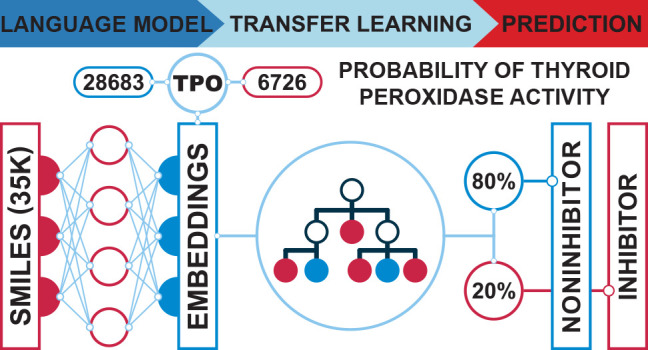

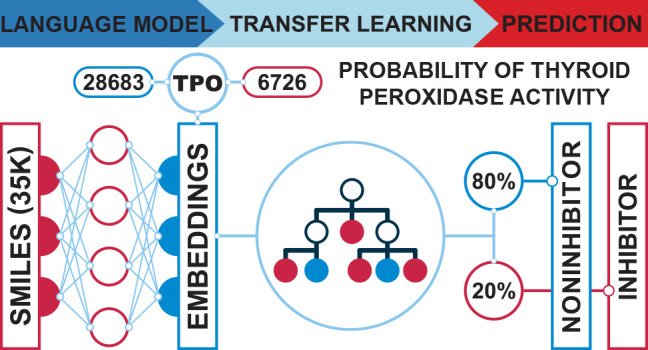

## 1 Introduction

The functioning of the human body is controlled by the endocrine system, which produces and regulates the release of various hormones. Disruption of this system can result from abnormal hormonal activity or exposure to endocrine-disrupting chemicals (EDCs).^1–4^ Contact with EDCs can result in a malfunctioning endocrine system, which may cause severe health disorders in humans and wildlife.^5^ Historically, the primary focus of EDC studies has been on estrogen and androgen receptors, although most nuclear receptors in humans are potential targets for EDCs.^6^ More recently, the scope of the research has broadened, and the focus has also included the thyroid hormone system.^7^

Depending on the developmental stage, thyroid hormones (THs) regulate many physiological processes in mammals and, consequently, may influence the structure and functioning of multiple organs. During pregnancy, THs are essential for embryonic brain and neural development,^8^ and especially in childhood, they play a critical role in the production of growth hormones and bone development.^9^ In adults, THs primarily regulate metabolism,^10^ and they also increase heart rate and cardiac contractility.^11^ Therefore, proper regulation and controlled release of the THs into the body are essential for maintaining physiological homeostasis.

THs are synthesized through a series of biochemical reactions (Figure 1) that take place within thyroid follicles.^12^ Iodine, thyroglobulin, thyroid peroxidase (TPO), and hydrogen peroxide are essential for successful TH biosynthesis. Thyroglobulin (Tg) serves as the main precursor to TH biosynthesis and provides intrathyroidal storage for iodine. Iodide is actively transported into the thyroid follicular cells by the Sodium/Iodide Symporter (NIS) and added to tyrosyl residues of Tg in the lumen with the help of TPO. Initially, TPO uses hydrogen peroxide to catalyze the oxidation of iodide to active iodine. Then, TPO incorporates the active iodine into the tyrosyl residues of Tg to form the TH precursors monoiodotyrosine (MIT) and diiodotyrosine (DIT)^13^ and modulates coupling of these precursors for the formation of tetraiodothyronine (T4) and triiodothyronine (T3).^14–16^ THs are released into the systemic circulation after proteolytic cleavage from Tg. The remaining MIT and DIT are subsequently deiodinated by iodotyrosine dehalogenase, recycling iodide, which is essential for TH synthesis.^12^ Inhibition of these steps can disrupt TH synthesis and availability, leading to direct thyroid toxicity such as thyroid hyperplasia/hypertrophy and a wide range of adverse outcomes including cognitive deficits affecting learning and memory, hearing loss, visual dysfunction and cardiovascular impairments.^17–19^

**Figure 1:**
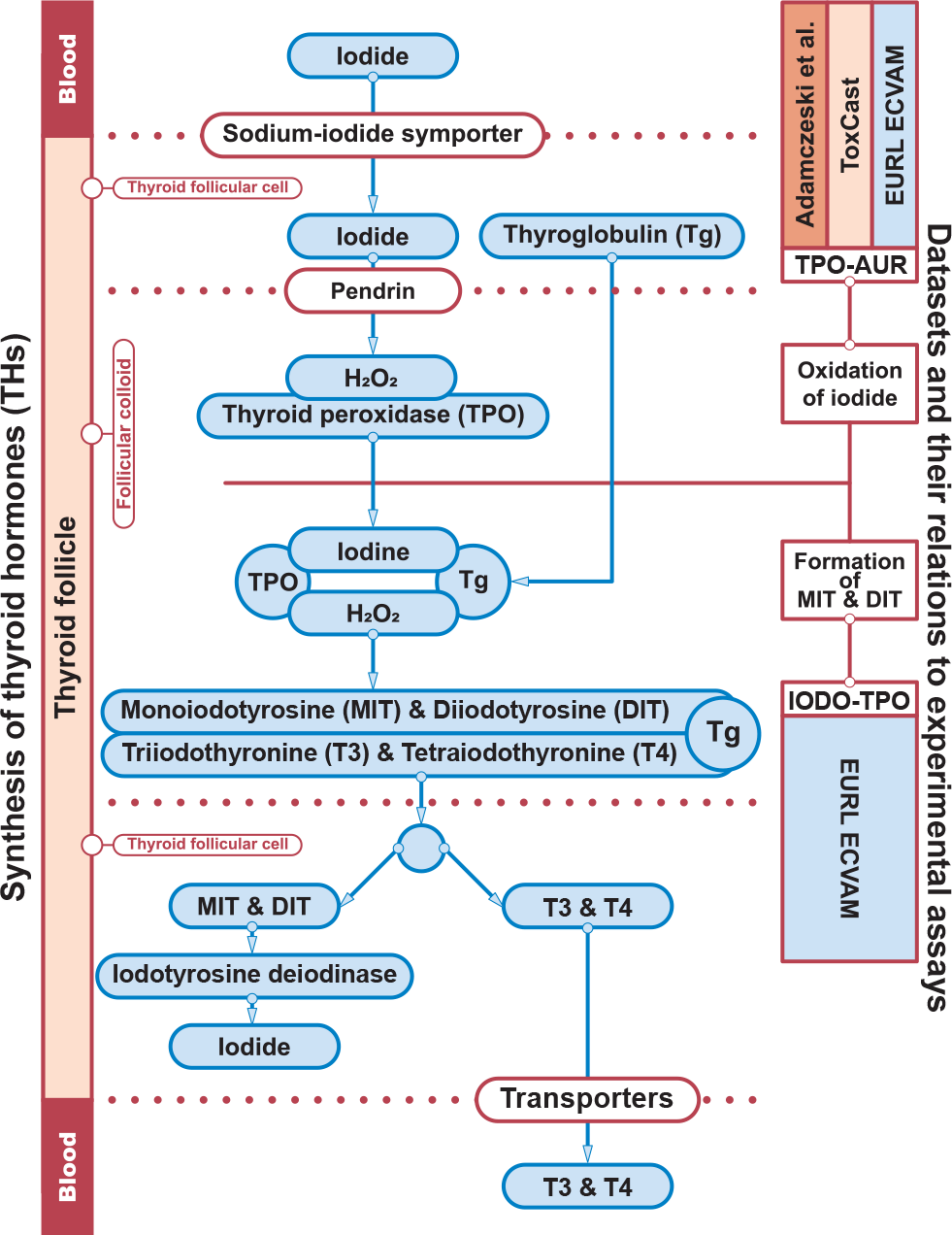
Synthesis of thyroidal hormones and relation of steps to experimental assays (data sets used in study).

TPO inhibition is a well-documented molecular initiating event (MIE) that is relevant across multiple species.^20^ Although it represents a critical regulatory endpoint for the identification of thyroid-disrupting chemicals, there are currently no validated OECD or US EPA guideline study protocols for its assessment. Low-throughput assays using indirect measurements are typically employed. Nevertheless, ongoing research and regulatory initiatives demonstrate substantial efforts to develop and validate novel assays to investigate this MIE.^21^ In this regard, the European Commission and Member States Partnership for the Assessment of Risks from Chemicals (PARC)^22^ has identified the lack of validated in silico and in vitro methods for assessing thyroid hormone system^23^ disruption as a major regulatory gap.

The use of chemicals for various purposes has increased during the past decades (demonstrated by the rise of analytical data in public databases^24^). Chemicals are primarily intended to benefit everyday life. However, some have been proven to pose risks to human health and the environment.^25^ Therefore, mitigating risks by conducting a range of experimental tests before introducing new chemicals is essential. However, testing all chemicals experimentally is not feasible. New approach methodologies (NAMs), such as high-throughput capable assays and computational models, can reduce the cost and time constraints associated with hazard identification. In this context, the use of efficient computational models to identify TPO inhibitors from large pool of chemicals represents a viable strategy for early detection of disruption of the thyroid hormone system.

Efforts to create computational NAMs for detecting endocrine-disrupting effects of chemicals have been ongoing for a long time. For example, collaborative modeling initiatives for predicting estrogen and androgen receptor activity have emerged. The U.S. Environmental Protection Agency (EPA) initiated the Collaborative Estrogen Receptor Activity Prediction Project (CERAPP), which focused on estrogen receptor (ER) activity,^26^ and the Collaborative Modeling Project for Androgen Receptor Activity (CoMPARA) focused on androgen receptor activity.^27^ Such considerable effort has not been made for thyroid system, although some models are available for predicting effects on thyroid-related endpoints.^28–32^ So far, models^33–36^ for TPO inhibition have been based on the ToxCast Phase I and II data sets, which originally included 1074 unique chemicals initially screened for TPO inhibition by measuring their oxidizing capacity at a single concentration using the Amplex UltraRed-thyroperoxidase (AUR-TPO) assay. Positive chemicals from the initial screen (≥20% decrease in TPO activity) were further evaluated for potentially low specificity using (i) concentration–response AUR-TPO assay, (ii) a cell-free luciferase assay for nonspecific enzyme inhibition, (iii) a cytotoxicity assay on a human cell line for cellular tolerance, and (iv) the guaiacol oxidation (GUA) assay for TPO inhibition.^37^ Recently, Adamczewski et al.^38^ released new models built on an extensive set of agrochemicals assessed for TPO inhibition as previously described.^37^ This data set is structurally more diverse than the previously available ToxCast data set. It creates new possibilities for models with a wider applicability domain, improved accuracy, and the ability to leverage various machine learning and artificial intelligence tools.

For decades, various machine learning tools have been used to develop predictive models within the framework of the quantitative structure–activity relationships (QSARs) approach. In such cases, the structures of small molecules are typically represented using molecular descriptors or chemical fingerprints and mapped to the properties/activities under study using different algorithms. The emergence of large language models (LLMs) offers new possibilities for molecule representations. However, general-purpose LLMs are not specifically designed to encode structural information from chemicals. For that, novel artificial intelligence (AI) approaches are being developed that represent chemical structures using pretrained embeddings.^39^ Training such specialized language models is usually unsupervised and based on much larger chemical data sets that are typically available for QSAR modeling. For example, MolBERT^40^ is trained on 1.27 million molecules using SMILES as the structural representation, and newer models such as Molformer^41^ and X-MOL^42^ already utilize 1.1 billion molecules for training. This makes embeddings information-rich and beneficial for modeling tasks when calculated for new molecules.

Screening large data sets for putative TPO inhibitors using computational models is a time- and cost-efficient approach for the early identification of chemicals of concern, that could support the prioritization of regulatory actions and ensure protection of human health and non-target organisms without unnecessary animal testing. This study aimed to evaluate the suitability of transfer learning from SMILES embeddings in comparison to classical molecular descriptors for the development of AI-driven QSAR models for the classification of TPO inhibitors.

## 2 Data and Methods

### 2.1 Thyroid Peroxidase Inhibition Data

The data used in this study came from two sources, and two distinct data sets were formed: a modeling set and an external validation set. The most extensive published agrochemicals data set with TPO inhibition data^38^ was selected as the modeling data set, and the ToxCast data set^36^ was used as the validation data set. Both data sets are derived from methods that specifically measure iodide oxidation inhibition. The initial model-building data set contained 34524 chemicals, and the ToxCast validation data set contained 1052 chemicals. Chemical structures were standardized using a QSAR-ready chemical structures preprocessing workflow^43^ that removed inorganics, organometallics, and mixtures from the data sets. The workflow also included stripping known salts, removal of stereochemistry, standardization of tautomers and nitro groups, correction of valences, and neutralization of structures when possible. Additionally, the data set was screened for duplicates. Duplicate structures were manually double-checked, and a single representative was retained when experimental values for all duplicates matched. If structures with conflicting values were observed, corresponding entries were removed from the data set. Finally, overlap between the two data sets was checked, and 16 duplicated compounds were removed from the validation set. This procedure resulted in 34391 chemicals for modeling and 1018 chemicals for external validation (validation set), represented as standardized SMILES.

The modeling data set was further divided into training and test sets. The training set was formed to address the imbalance in the modeling set, which included 6481 inhibitors and 27910 noninhibitors. The training set was constructed by random under-sampling an equal number of TPO inhibitors and noninhibitors (corresponding to 80% of the minority class). All the remaining chemicals were used as a test set. The same approach was successfully applied in modeling androgen receptor activity^44^ and estrogen receptor activity.^45^ Table 1 shows the distribution between TPO inhibitors and noninhibitors for all data sets.

**Table 1:** Distribution of Inhibitors and Noninhibitors in the Datasets

Set	Total	Inhibitor	Noninhibitor
Training set	10368	5184	5184
Test set	24023	1297	22726
Validation set	1018	245	773

To assess the predictive performance of the model, data from the European Food Safety Authority (EFSA) assessments for the identification of pesticide active substances with endocrine disrupting properties (ED assessments)^46^ and data generated by the European Union Network of Laboratories for the validation of alternative methods (EU-NETVAL) during a validation study coordinated by the European Union Reference Laboratory for Alternatives to Animal Testing (EURL ECVAM)^47,48^ were used (see Section 3.5 for more details).

### 2.2 Molecular Descriptors of Structure Representation

Standardizing SMILES is necessary for reproducible descriptor values. Therefore, standardized SMILES structures were used as inputs to characterize chemicals numerically using two fundamentally different methods. The first approach used theoretical molecular descriptors calculated with DRAGON software (version 6.0.38).^49^ Since geometric descriptors (3D) are not easily reproduced and require optimization of structure, all 1D and 2D descriptors (4885) were calculated. The second approach used a pretrained deep neural network model or specialized language model. Among many available options, MolBERT^40^ was chosen for its ease of integration into established workflows. MolBERT is a bidirectional language model that utilizes the Bidirectional Encoder Representations from Transformers (BERT) architecture^50^ and transforms structural information and nuances from SMILES into a 768-dimensional numerical vector. Those vectors are known as SMILES embeddings, and in some sense, they can be viewed as a new type of molecular descriptor because a deep artificial neural network encodes SMILES structures into numerical feature vectors that capture information about molecular constitution (atoms, their properties, and connectivity). The embeddings were extracted from the final MolBERT model (pretrained using 100 epochs) without any modifications.

### 2.3 Similarity Analysis Methods

Fingerprint-based similarity analyses mostly rely on the analysis of similarity/distance matrices, where similar chemicals have the smallest distance between them or the highest similarity score. Analyzing these matrices can be difficult, especially as the data set grows. It is feasible to analyze these matrices on smaller data sets as heat maps. Regardless, it has been considered advantageous to plot fingerprint-based similarity on larger data sets using the Rubberbanding Force field approach built into the DataWarrior^51^ software (version 06.04.02). By default, DataWarrior uses a custom FragFP fingerprint for analysis. It is a binary fingerprint that consists of 512 predefined substructure fragments. These fragments occur frequently in organic chemicals and exhibit minimal overlap within a set of organic chemicals. In the similarity analysis, chemicals were grouped based on the entire similarity matrix, and their locations on a 2-D scatter plot were optimized to establish connections between similar compounds.

Analysis of calculated molecular descriptors can also give a similarity estimate of chemicals, where, in theory, chemicals with closer descriptor values are more similar. Usually, hundreds or even thousands of descriptors are calculated for each chemical, making the visualization of such a high-dimensional descriptor space very difficult. There are many dimension reduction methods, but here the t-distributed stochastic neighbor embedding (t-SNE)^52^ was used to visualize high-dimensional variable space. The t-SNE method assigns for each data point a location on a two-dimensional map, grouping data points that are more similar to each other.

### 2.4 Model Development Components

#### 2.4.1 Random Forest Algorithm

The Random Forest (RF)^53^ algorithm implemented in the randomForest package (version 4.7–1.2) and the R statistical software (version 4.5.1)^54^ were used for the classification analysis. The RF algorithm is based on an ensemble of decision trees and can handle classification and regression problems. In classification, the consensus of all the trees (the majority vote) is used to make the final decision. The model is trained by growing each tree using a random sample of two-thirds of the chemicals from the data set (bootstrap sample). The chemicals that do not participate in the tree-growing process serve as an internal validation set to estimate the model’s error and are referred to as out-of-bag (OOB) samples. Each tree is grown by selecting the best descriptors from a random subset of descriptors. In the current work, the random sample size was equal to the square root of the total number of descriptors. Each tree was grown until splitting was impossible. All statistical parameters in the tables for the training set correspond to the OOB predictions.

#### 2.4.2 Selection of Descriptors

The selection of descriptors began with a preprocessing step that removed descriptors with missing values and those with zero variance. For full models, all the remaining descriptors were used for model building. Selecting descriptors for the reduced model was an iterative procedure that ranked the variable importance of descriptors from the RF algorithm. The variable importance analysis performs a permutation test for each descriptor and estimates its importance using the mean decrease in accuracy. It randomly shuffles descriptor values and uses OOB compounds to calculate the reduction in accuracy compared to the original unshuffled data.

The most useful descriptors were preselected by building 100 models with randomly split data sets. The three most important descriptors were selected from each model, and unique descriptors were included in the descriptor pool for the next iteration. This procedure was repeated until no further changes were observed in the unique descriptors, and the final model was constructed using these descriptors.

### 2.5 Model Performance Metrics

The performance of classification models was assessed based on the number of true positive (TP), true negative (TN), false positive (FP), and false negative (FN) predictions, which form the confusion matrix. They are also used to derive different metrics to analyze the quality of the models from various angles. This work analyses models with sensitivity (eq 1), specificity (eq 2), accuracy (eq 3), balanced accuracy (eq 4), and the Matthews correlation coefficient (MCC, eq 5). Positive predictions correspond to the TPO inhibitors, and negative predictions correspond to the noninhibitors. Therefore, sensitivity shows the proportion of correctly classified inhibitors, specificity measures the proportion of correctly classified noninhibitors, and accuracy corresponds to the ratio of correctly classified chemicals. The classes in the studied data series were not initially balanced. Therefore, the majority class may be responsible for overoptimistic accuracy estimation.^55,56^ Balanced accuracy and MCC (ranging from −1, representing inverse prediction, to 1, indicating perfect prediction) were also used to assess model quality and identify potential issues related to class imbalance.

1 $$Sensitivity=\frac{TP}{TP+FN}$$

2 $$Specificity=\frac{TN}{TN+FP}$$

3 $$Accuracy=\frac{TP+TN}{TP+TN+FP+FN}$$

4 $$Balanced\;accuracy=\frac{Sensitivity+Specificity}{2}$$

5 $$MCC=\frac{TP\times TN-FP\times FN}{\sqrt{\left(TP+FP\right)\left(TP+FN\right)\left(TN+FP\right)\left(TN+FN\right)}}$$

### 2.6 Availability of Model

Best practices for QSAR model reporting^57^ suggest that QSARs and related data can be made available in various formats.^24^ To follow best practices, the QSAR Data Bank format^58^ is used, and models with data are stored in the QsarDB repository,^59,60^ as in our recent example.^61^ A digital object identifier (DOI) is assigned for the models and data.^62^

## 3 Results and Discussions

### 3.1 Models for Different Molecular Descriptors

Successful QSAR modeling depends on many aspects. Among them are selecting an appropriate set of molecular descriptors and properly preprocessing the data set. This study primarily focused on comparing the performance of traditional molecular descriptors calculated with the DRAGON software and SMILES embeddings generated by the MolBERT deep neural network model. Independent of the modeling algorithm, it is known that class imbalance can introduce bias in the development of classification models.^63^ The preliminary analysis revealed that, despite the data set being large compared to a typical QSAR data set, it still led to majority class bias in the prediction results. Meaning that the model derived from an imbalanced data set failed to predict the minority class. Therefore, all the models in the current study were derived using randomly undersampled data sets.

Modeling TPO inhibition using two different molecular structure representations gave varying results. The model that uses MolBERT descriptors is approximately 4% more accurate. There are no significant differences in statistical parameters between the training and test sets (see Table 2). The most significant difference between these models becomes apparent when compared with an external validation set. Both models can correctly identify noninhibitors reasonably well (specificity: DRAGON, 0.742; MolBERT, 0.772), but the DRAGON-based model fails to detect inhibitors (sensitivity, 0.490). On the other hand, the model using SMILES embeddings detects inhibitors and noninhibitors equally well (sensitivity 0.788, specificity 0.772), and the MCC (0.498) also confirms this. This raises the question about the reason for such a difference and whether it could be explained by a more detailed analysis of the chemical space of the data sets (see the next section).

**Table 2:** Results from Models Using All the Molecular Descriptors/Representations

	Descriptors from DRAGON			Embeddings from MolBERT		
Performance	Training	Test	Validation	Training	Test	Validation
Accuracy	0.785	0.767	0.681	0.824	0.802	0.776
Sensitivity	0.809	0.819	0.490	0.855	0.848	0.788
Specificity	0.761	0.764	0.742	0.793	0.8	0.772
Balanced accuracy	0.785	0.791	0.616	0.824	0.824	0.780
MCC	0.571	0.297	0.213	0.649	0.345	0.498

### 3.2 Analysis of Chemical Space

Comparing modeling results across different types of descriptors suggests that traditional molecular descriptors may not capture the full chemical diversity of both data sets. Adamczewski et al.^38^ performed a thorough analysis of structural diversity between the agrochemical (modeling set) and ToxCast (validation set) data sets. They compared differences between physicochemical properties, Bemis–Murcko scaffold diversity and structural features. All of them showed significant differences between the two data sets, with the most pronounced differences observed in ring structures, particularly heteroaromatic rings. To understand the differences between data sets and to examine the discriminative power of the descriptors, data sets were analyzed using fingerprints, molecular descriptors, and SMILES embeddings.

The densely packed structural map (Figure 2) obtained from comparative similarity analysis based on fingerprints contains the following information. Each point corresponds to one chemical. Squared points represent TPO inhibitors, and circular points represent noninhibitors. Chemicals from the modeling set are colored blue, and those from the ToxCast set are colored red. Points in the middle of the figure are connected and form clusters of compounds that are more similar to each other. Chemicals in the outer ring are less similar to each other. Several trends can be observed that support the hypothesis that the two data sets are not structurally similar. First, most of the ToxCast chemicals overlap very little with the densest region of the modeling data set, only a few tiny clusters, as highlighted in Figure 2A (chemicals connected with purple lines). This means that there are no compounds with such structural patterns among the ToxCast compounds, indicating no similarity in this part of the structural space. Second, if compounds form a close cluster, they usually form it among themselves in the modeling set (Figure 2B and C). Only very few clusters are formed inside the validation set (Figure 2D). This means they are more similar within a set than the chemicals in another set (i.e., in the modeling set). The third observation is that many chemicals have virtually no associations with other chemicals in the data set (Figure 2E); that is, there are many chemicals with unique structural features in both data sets. From the above, it can be concluded that the modeling set contains many structurally similar compounds and a number of unique structures, which suggests that the models developed on the data set have a potentially broad applicability domain. Furthermore, the fingerprint (ECFP4, 2048-bit) Tanimoto similarity comparison between the validation and modeling sets showed that 4.1% of compounds in the validation set had a similarity greater than 0.6, and only 0.9% had a similarity greater than 0.7. This shows that MolBERT-based models can extrapolate beyond the chemical space in which they were trained.

**Figure 2:**
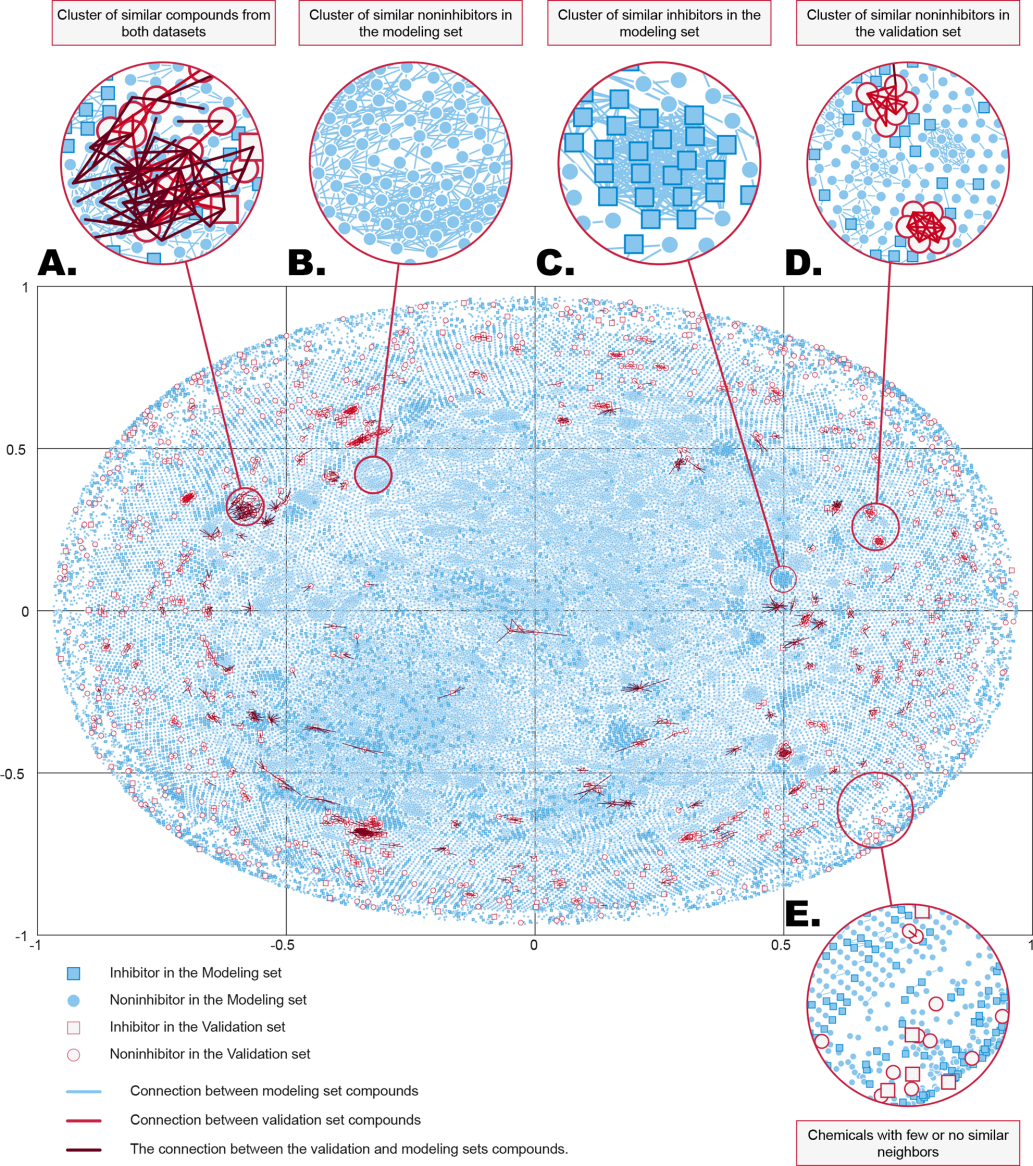
Chemical neighborhood similarity map using DataWarrior Rubberbanding Force field and FragFP fingerprints.

The descriptor space analysis, using t-SNE plots (Figures 3 and 4), provides insight into why the model with traditional descriptors was unable to predict structures that were more different from the compounds in the modeling set. It is known that these plots are sensitive to hyperparameters. Furthermore, compressing information from hundreds of descriptors into a two-dimensional space causes some information loss, and the interpretation of these plots is somewhat subjective. However, a preliminary analysis of the plots from multiple runs showed that the distribution of chemicals by molecular weight remained similar. For the DRAGON descriptors, there was a clear size dependence, meaning that larger compounds were mostly at one end of the plot and smaller ones at the other. In contrast, for SMILES embeddings, compounds of different sizes were evenly distributed across the plot. At the same time, both descriptor sets appear to cluster within regions in the modeling set that are predominantly populated with noninhibitors. In the case of DRAGON descriptors, many more inhibitors from the validation set occupy areas where the modeling set has mostly noninhibitors. In the case of MolBERT SMILES embeddings, inhibitors and noninhibitors from the validation set appear more coherently placed in their respective regions relative to the modeling set. One possible reason to hypothesize might be that SMILES embeddings capture more structural nuances than traditional descriptors. Therefore, they do not fail when structurally different chemicals are used for prediction.

**Figure 3:**
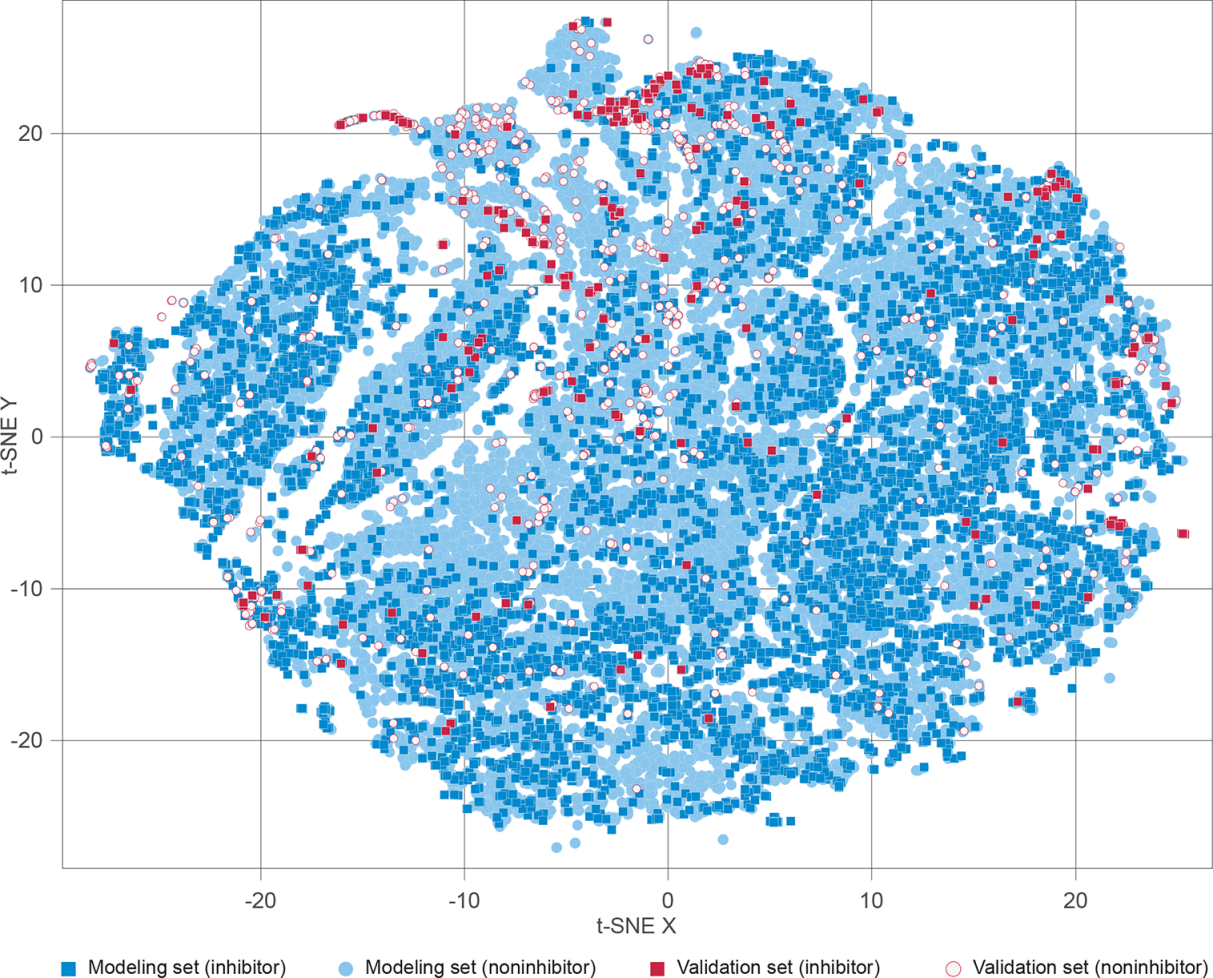
t-SNE plot of DRAGON descriptors.

**Figure 4:**
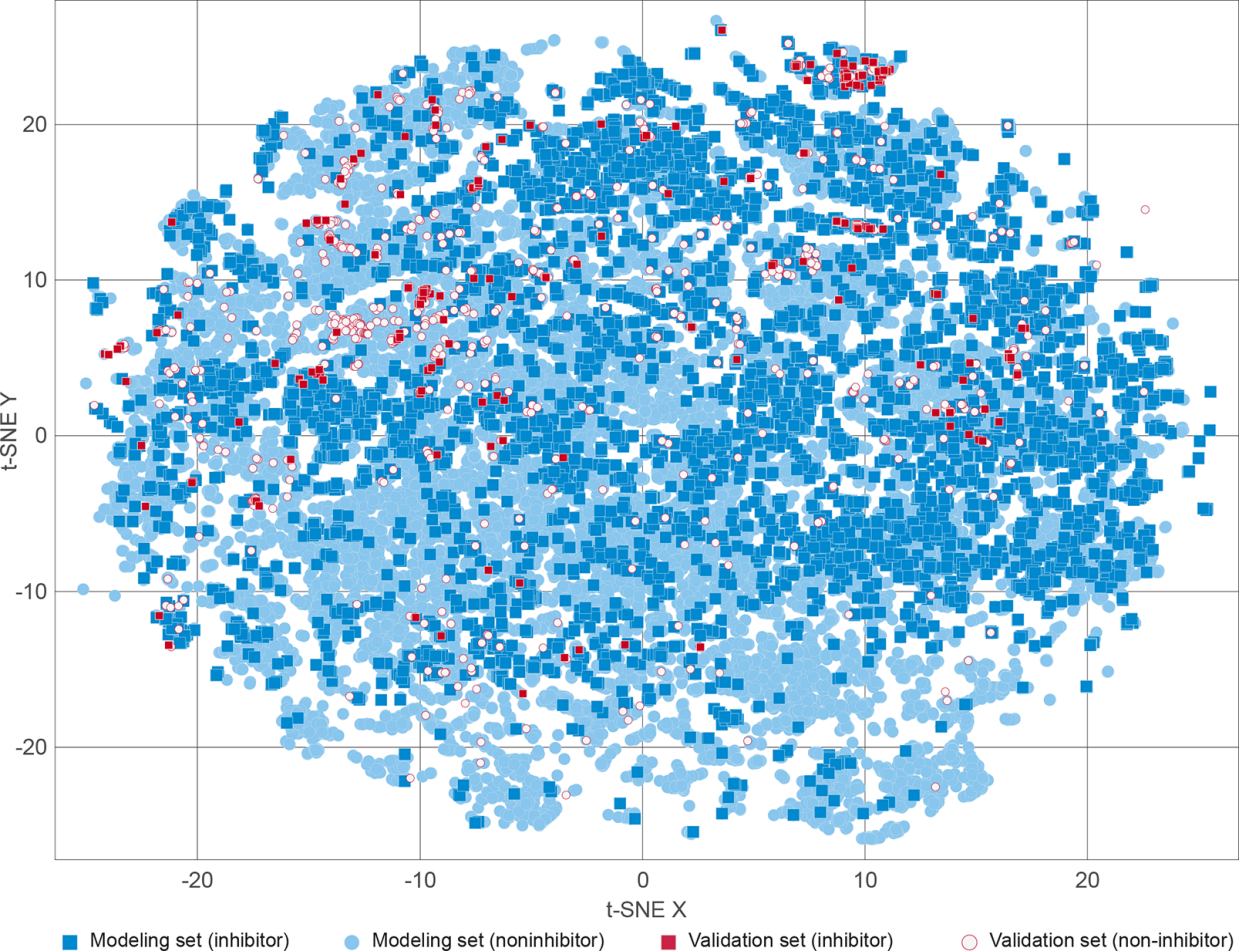
t-SNE plot of MolBERT SMILES embeddings.

### 3.3 Optimization of the Final Model

When building machine learning QSAR models, using as much information as possible can be beneficial but it may lead to overfitting. Since the RF algorithm employs random sampling of variables, it is relatively immune to overfitting. However, in QSAR type modeling, it is often best to use as simple a model as possible and as complex a model as necessary. Making the RF model simpler can be achieved in two ways. First, reducing the number of variables can make models more interpretable. Second, reducing the number of trees in the forest.

Molecular representations from MolBERT are challenging to interpret. However, there was a desire to test whether reducing the variable space negatively affects the model’s performance on the external validation set. In other words, how much structural variability is encoded into a small number of variables? After the variable reduction procedure, a predictive model with only five variables was achieved. As expected, the model performed worse than the model using all MolBERT representations, but model training and test set performance were still comparable to those of the model with DRAGON descriptors (Tables 2 and 3). It turned out that the model retained its ability to identify both inhibitors and noninhibitors equally well. All statistical parameters remained uniform across data sets. It suggests that each SMILES embedding is sufficiently rich in structural information.

**Table 3:** Results for a Model with Reduced Variable Space

	Representations from MolBERT (reduced)		
Performance	Training	Test	Validation
Accuracy	0.736	0.745	0.71
Sensitivity	0.734	0.763	0.739
Specificity	0.738	0.744	0.702
Balanced accuracy	0.736	0.753	0.720
MCC	0.472	0.254	0.386

To compare the effect of forest size on model quality, seven models with different forest sizes were built using the same training set. Even though each data set suggests a different model as the best one (Table 4, bold), the statistical parameters for these models were similar. For example, the difference in Balanced Accuracy between the smallest and the largest models on the test and validation sets was only 0.009 and 0.019, respectively. The smaller model performs as well as the larger ones. Therefore, given the trade-off between model performance and computational cost during training, the small increase in performance of the larger models is not sufficient to justify choosing them over the smallest one.

**Table 4:** Statistical Parameters for Different-Sized Models

		Number of trees (model size)						
	Performance	51	101	201	301	401	501	1001
Training	Accuracy	0.799	0.812	0.818	0.822	0.823	0.824	**0.827**
	Sensitivity	0.811	0.836	0.847	0.851	0.854	0.855	**0.861**
	Specificity	0.786	0.789	0.788	0.792	0.792	**0.793**	**0.793**
	Bal. accuracy	0.799	0.812	0.818	0.822	0.823	0.824	**0.827**
Test	Accuracy	0.793	0.797	0.80	0.801	0.801	**0.802**	**0.802**
	Sensitivity	0.838	0.843	**0.850**	0.844	0.848	0.848	0.847
	Specificity	0.790	0.795	0.797	0.799	0.798	**0.80**	**0.80**
	Bal. accuracy	0.814	0.819	0.823	0.821	0.823	**0.824**	0.823
Validation	Accuracy	0.753	0.759	0.772	0.776	**0.779**	0.776	0.772
	Sensitivity	0.763	0.776	**0.788**	**0.788**	**0.788**	**0.788**	0.784
	Specificity	0.750	0.754	0.767	0.772	**0.776**	0.772	0.768
	Bal. accuracy	0.757	0.765	0.777	0.780	**0.782**	0.780	0.776

Table 5, together with Table 6, confirms that despite an imbalanced test and validation set, the chosen model’s statistical parameters are close to those of a balanced training set. Additionally, summarized statistical parameters (Table 6) from 100 models are very similar to the statistics of the final model, demonstrating that the final model is robust and not affected by sampling bias.

**Table 5:** Confusion Matrices for the Final 51-Tree Model

			Predicted TPO	
			Inhibitor	Noninhibitor
Experimental TPO	Training	Inhibitor	4202	982
		Noninhibitor	1107	4077
	Test	Inhibitor	1087	210
		Noninhibitor	4769	17957
	Validation	Inhibitor	187	58
		Noninhibitor	193	580

**Table 6:** Statistical Parameters for the Final Model, and Summarised Statistics over 100 Models with Different Splits

	Statistic	Final model	Min	Max	Average	Std. deviation
Training	Accuracy	0.799	0.788	0.806	0.799	0.004
	Sensitivity	0.811	0.797	0.823	0.813	0.005
	Specificity	0.786	0.770	0.801	0.785	0.005
	Bal. accuracy	0.799	0.788	0.806	0.799	0.004
	MCC	0.597	0.576	0.613	0.598	0.008
	PR-AUC	0.871	0.859	0.881	0.871	0.004
Test	Accuracy	0.793	0.782	0.806	0.795	0.005
	Sensitivity	0.838	0.821	0.866	0.841	0.010
	Specificity	0.790	0.779	0.804	0.792	0.005
	Bal. accuracy	0.814	0.806	0.827	0.817	0.005
	MCC	0.331	0.317	0.347	0.334	0.006
	PR-AUC	0.439	0.399	0.464	0.429	0.014
Validation	Accuracy	0.753	0.727	0.781	0.754	0.011
	Sensitivity	0.763	0.673	0.800	0.737	0.028
	Specificity	0.750	0.728	0.794	0.759	0.013
	Bal. accuracy	0.757	0.712	0.787	0.748	0.015
	MCC	0.454	0.379	0.511	0.442	0.025
	PR-AUC	0.623	0.548	0.641	0.596	0.021

### 3.4 Interpretability of Final Model

When examining the interpretability of different types of descriptors, one can identify disadvantages in using SMILES embeddings. Compared to traditional molecular descriptors, the interpretation of embeddings is difficult. For example, the most important descriptor (see Supporting Information Table S3 for full list) in the DRAGON model is NdsN (Number of atoms of type dsN). For the final model from MolBERT descriptors, it is the embedding number 142 (E142). The NdsN is connected to the imine functional group, and hydroxyl imines were among novel substructures identified as potential TPO inhibitors.^38^ Therefore, this kind of information can be associated with TPO inhibition. However, the embedding E142 has no direct chemical explanation, i.e., it is not interpretable according to current knowledge. To enhance the interpretability of machine learning models, typically, SHAP analysis^64^ is used. For the SMILES embeddings, SHAP analysis provides information on which embedding (descriptor) is more important and how its value influences the property, but it does not help interpret the meaning of the embedding itself. Therefore, connecting the structural information encoded in the embedding (structure–activity relationship) with the mechanistic information on the property (TPO inhibition) remains unsolved at present due to difficulties in explaining the link between chemical structure and the information coded in the embedding. Overall, the results from transfer learning offer better predictive power than the traditional approach. For further analysis, the final model was also compared against chemicals of interest assessed by EFSA and EURL ECVAM.

### 3.5 Assessment of External Data Sets

The final 51-tree model (Table 5) was used to predict TPO inhibition in (a) selected agrochemical substances for which TPO experimental data were available in the EFSA endocrine disruption assessments concluded by December 2025 in accordance with Commission Regulation (EU) 2018/605 ^46,65^ (see Supporting Information Table S1) and (b) a list of substances that have been tested experimentally by the European Union Network of Laboratories for the Validation of Alternative Methods (EU-NETVAL) test laboratories (TLs) as part of the Thyroid Validation Study (TVS) initiated by EURL ECVAM^47^ (see Supporting Information Table S2).

In EFSA endocrine disruption assessments that are concluded and are publicly available, data on TPO activity have mostly been retrieved from the USEPA Comptox Dashboard^66^ and therefore could not be used to further assess model predictivity, as ToxCast data are part of the validation data set. Thus, from the EFSA data set, only 8 substances with available TPO experimental data were considered, which is a limited sample size for a conclusive assessment of model predictivity. It is also acknowledged that in the absence of validated protocols on TPO inhibition, the methods used relied on different in-house protocols, and the EFSA peer review outcomes have been considered without reevaluation. None of these chemicals were included in the modeling set. Thiophanate-methyl and Thiabendazole were part of the validation set based on ToxCast data. Thiophanate-methyl was positive based on in vitro TPO experimental data in the EFSA assessment and was also predicted to be TPO inhibitor by the model. Benthiavalicarb, Pydiflumetofen, Lenacil, Clodinafop-propargyl, and Thiabendazole, with negative TPO experimental data, were also predicted to be noninhibitors using the model. Fludioxonil and Proquinazid were predicted to be TPO inhibitors, whereas experimental data showed the opposite.

It is well recognized that several molecular targets can disrupt the thyroid hormone system.^17^ Although TPO inhibition may cause changes in thyroid histopathology, assessment of this MIE as recommended in the EFSA/ECHA/JRC Guidance,^67^ addresses endocrine activity only in part, as other mechanisms may also plausibly lead to thyroid-related adversity. For example, although Benthiavalicarb and Thiabendazole were concluded to be thyroid hormone system disruptors based on evidence on thyroid adversity from in vivo experimental data, they were considered as TPO noninhibitors in in vitro data. This finding should not be interpreted as reflecting low predictivity of an in silico or in vitro model, but rather as an indication of the involvement of additional mechanisms and the inherent complexity of biological systems.

Two distinct TPO assays were developed and implemented by the European Union Network of Laboratories for the Validation of Alternative Methods (EU-NETVAL) test laboratories (TLs) as part of the Thyroid Validation Study (TVS) initiated by EURL ECVAM.^68^ The TPO-AUR method^69^ targets the oxidative capacity of TPO, while the TPO-IODO assay^70^ quantifies both TPO oxidative capacity and the TPO-catalyzed formation of monoiodotyrosine (MIT), a precursor to THs. Thirty coded chemicals were selected by a panel of topic experts together with EURL ECVAM. In addition, 1 chemical tested in both methods, and 2 chemicals tested with the TPO-IODO method were included, to derive a total of 33 chemicals with experimental data. For this study, three inorganic compounds were excluded because they could not be predicted with the proposed model, and three other chemicals were excluded because they were also used to develop the predictive model. The inhibition of TPO (see Supporting Information Table S2) was tested experimentally for this set of test chemicals by using two distinct methodologies: (1) change in fluorescence when AUR- is oxidized by TPO (TPO-AUR) and (2) quantification of MIT formation using LC-MS/MS when TPO iodinates tyrosine (TPO-IODO). For both methods, the test system consisted of cell lysates from FTC-238 hTPO cells, a cell line derived from lung metastases of a human follicular thyroid carcinoma that had been transfected with human recombinant TPO.

The comparison of results from the 25 chemicals tested in both assays (see Supporting Information Table S2) reveals that 11 out of 25 chemicals were identified as positive (inhibitors) with both the TPO-AUR assay and TPO-IODO assay. The TPO-IODO assay identified additional 11 chemicals as positive, so a total of 22 out of 25 chemicals. The latter 11 chemicals were identified as negative in the TPO-AUR assay (noninhibitors). The differences appear to be primarily attributable to mechanistic differences, as TPO-IODO can detect disruptors of the iodination activity of TPO in addition to inhibitors of oxidative capacity, but also due to the lower sensitivity of the AUR method. Since the modeling data set was based on iodide oxidation inhibition, the model was compared with the TPO-AUR assay results, even though the TPO-IODO assay was considered a more sensitive and reliable method for detecting TPO disruptors.

Of the 25 chemicals with AUR experimental data, 11 were also present in the validation data set but are nevertheless discussed in this section. Out of 11 inhibitors (based on the AUR test), the model correctly predicted 9 (see Supporting Information, Table S2). In contrast, two were incorrectly predicted as noninhibitors, namely Pentachlorophenol and TETRAC. However, TETRAC was also present in a validation set and was considered a noninhibitor there. Out of 14 AUR noninhibitors, only 5 were correctly predicted by the model. One of them, Ketoconazole, was in the validation set and was considered an inhibitor. Interestingly, most of the misclassified noninhibitors by the model were identified as inhibitors with the IODO assay. The only exception was Amiodarone, which was a noninhibitor in the validation set and tested as a noninhibitor in both assays, yet was predicted as an inhibitor by the model. It is known that Amiodarone is a highly hydrophobic compound (LogKow > 4.5) and binds strongly to proteins and plasticware.^47^ It would be worthwhile to investigate the reasons for these discrepancies further.

## 4 Conclusions

A large and diverse data set of 34391 agrochemicals was used to derive classification models for predicting the TPO inhibition activity. The study focused on improving the predictive ability and the applicability of classification models for TPO inhibitors. The most predictive models were obtained using a transfer learning approach, where SMILES embeddings generated by a pretrained deep neural network (MolBERT) were used to classify TPO inhibition activity in Random Forest models. This transfer learning approach was compared with a traditional machine learning approach, where Random Forest models were trained on a large set of theoretical molecular descriptors (DRAGON). The comparison of models showed that, regardless of the molecular representations used, the models produced similar results when validated with similar chemicals included in the modeling data. However, a considerable improvement in classification performance was observed for the ToxCast validation data set, despite the fact that this data set was structurally less similar compared to the training data. The model with theoretical descriptors was not able to identify TPO inhibitors with sufficient accuracy in the validation set. The final model, which used MolBERT SMILES embeddings, was able to identify both inhibitors and noninhibitors equally well in the validation data set, regardless of chemical similarity. This suggests that SMILES embeddings can characterize a much richer chemical space and thus expand the applicability of the models. The final model and underlying data are available in the QsarDB repository and follow FAIR principles.

A limited number of compounds evaluated by EFSA could be used to assess model predictivity, as ED assessments primarily relied on ToxCast data. The use of nonvalidated in vitro tests in regulatory assessments highlights the need for reliable and well-characterized TPO assays. Thus, the predictions of the 51-tree model showed higher sensitivity (approximately 70%) compared to the experimental data of the EURL ECVAM study on the tyrosine iodination method (TPO-IODO), reflecting on the complexity of biological systems. TPO catalyzes the oxidation of iodide to iodine, the transfer of active iodine to tyrosine residues, the formation of mono- (MIT) and di- (DIT) iodotyrosines, and the coupling of these precursors to form T3 and T4. Thus, inhibitors of downstream events, such as compounds interfering with the iodination step, may also be tested or predicted to be positive in the early oxidation steps. This outcome is expected to be chemical-specific and rely on the structural features present, like aromatic or halogenated rings and iodine acceptors. Analysis of the chemical space of the training set used in the development of the model has revealed a broad applicability domain with a number of unique structures, enhanced in ring structures, particularly heteroaromatic rings. As the validation process of in vitro TPO assays progresses, additional experimental data will help update and improve the computational models, including the 51-tree model.

## Supplementary Material



## Data Availability

Data and final 51-tree model are available at QsarDB.org repository (DOI: 10.15152/QDB.272), see reference 62 for complete citation of model and data source.

## Notes

List of agrochemical substances with TPO experimental data considered by EFSA in the endocrine disruption assessment (Table S1); list of chemicals selected by a panel of topic experts together with EURL-ECVAM (Table S2); DRAGON descriptors used for modeling, ordered by importance measure (mean decrease in accuracy) (Table S3) (XLSX)
